# ELK3-CYFIP2 axis-mediated actin remodeling modulates metastasis and natural killer cell responses in triple-negative breast cancer

**DOI:** 10.1186/s13046-025-03309-7

**Published:** 2025-02-10

**Authors:** Seung Hee Choi, Hye Jung Jang, Joo Dong Park, Ki Seo Ryu, Eunchong Maeng, Seohyun Cho, Hail Park, Hae-Yun Jung, Kyung-Soon Park

**Affiliations:** 1 Department of Biomedical Science, CHA University, Seongnam, Republic of Korea; 2 Department of Integrative Biotechnology, Sungkyunkwan University, Suwon, Republic of Korea; 3 Division of Radiation Biomedical Research, Korea Institute of Radiological and Medical Sciences, Seoul, Republic of Korea

**Keywords:** Triple-negative breast cancer, ELK3, CYFIP2, Filopodia, Actin accumulation, Natural killer cell

## Abstract

Triple-negative breast cancer (TNBC) is an aggressive, highly metastatic disease with a poor prognosis. E26 transformation-specific transcription factor (ELK3) is highly expressed in TNBCs, and functions as a regulator of epithelial-mesenchymal transition and immune responses. Because metastatic migration and immune evasion by TNBC cells are critical factors for successful metastasis, unravelling the underlying mechanisms and developing effective immunotherapeutic strategies is urgent. Here, TNBC cell lines MDA-MB-231 and Hs578T were examined to determine the relationship between ELK3 expression and filopodia protrusion on the cell membrane, as well as actin accumulation at contact sites with natural killer (NK) cells. RNA-sequencing analysis and molecular experiments were conducted to identify and validate downstream target genes of ELK3 associated with migration and attachment of TNBC cells. The immune response of TNBC to NK cells was evaluated through imaging and flow cytometry analyses. Clinical significance was assessed through Kaplan–Meier analysis of survival outcomes of TNBC patients. Gene expression profiling and molecular analysis revealed that oncogenic ELK3 directly suppresses expression of cytoplasmic FMR1 interacting protein2 (CYFIP2), a repressor of actin accumulation. Further molecular and pharmacological analyses confirmed that the ELK3-CYFIP2 axis serves a dual role in TNBC cell lines by (1) controlling filopodia-mediated migration and adhesion by regulating actin accumulation, and (2) regulating sensitivity to NK cells by modulating actin accumulation at contact sites. Kaplan–Meier analysis suggested that ELK3-CYFIP2 axis is associated with survival of TNBC patients, and that ELK3 suppresses transcription of CYFIP2. Thus, the ELK3-CYFIP2 axis plays a pivotal role in regulating actin, emphasizing its significance in controlling both cancer cell migration and NK cell responses in TNBC.

**Supplementary Information:**

The online version contains supplementary material available at 10.1186/s13046-025-03309-7.

## Introduction

Triple-negative breast cancer (TNBC) is a subtype of breast cancer distinguished by the lack of estrogen receptors, progesterone receptors, and human epidermal growth factor receptors 2 [1]. Although TNBC is sensitive to chemotherapy, patients with this subtype face increased mortality rates and poorer cause-specific and overall survival if a complete response is not attained [2–4]. This apparent discrepancy between chemotherapy responsiveness and survival outcomes underscores the urgent need for novel and more effective therapeutics. Natural killer (NK) cells are crucial for immunosurveillance, and for controlling cancer progression and metastasis [5]. The presence of NK cell infiltrates is associated with favorable prognosis for diverse solid tumors, including melanoma, gastric cancer, and breast cancer [6–9]. Thus, NK cell-based immunotherapy offers promise as a potential therapeutic avenue for TNBC patients.

The immune response of metastatic mesenchymal cancer cells to NK cells is controversial. For instance, the process of epithelial-mesenchymal transition (EMT) increases the susceptibility of cancer cells to NK cell-mediated cytotoxicity by altering expression of activating and inhibitory ligands [10]. Additionally, myocardin-related transcription factors, known for promoting metastatic invasion of melanoma and breast cancer cells, enhance cancer cell sensitivity to cytotoxic lymphocytes by increasing rigidity [11]. On the contrary, when TNBC cell lines were genetically modified to display epithelial cell morphology and reduced metastatic potential through suppression of oncogenic ELK3 expression, their sensitivity to NK cells increased [12]. These compelling lines of evidence indicate that there are numerous unaccounted-for variables affecting the immune sensitivity of metastatic cancer cells to NK cells.

Malignant cancer cells utilize their inherent migratory ability to invade neighboring tissues and vasculature, ultimately resulting in metastasis. The process of cell migration involves a sequence of steps initiated by formation of membrane protrusions. The driving force behind these protrusions is localized dynamic polymerization of submembrane actin filaments. Actin polymerization dynamics not only serve as the driving force for migration and metastasis of cancer cells, but also play a crucial role in mediating tumor immune evasion during cytotoxic lymphocyte attacks. This occurs through modulation of immune synapse formation at the interaction site [13, 14]. Specifically, breast cancer cells that are inherently resistant to NK cell-mediated killing exhibit significant actin accumulation at the contact site with immune cells [13]. While these reports collectively indicate an association between the metastatic behavior and immune response of cancer cells in terms of actin polymerization dynamics, little attention has been paid to the master mechanisms that regulate these phenotypes simultaneously.

WASP family verprolin homologous protein (WAVE), which is present in all eukaryotic kingdoms and plays a central role in cell division, fusion, adhesion, and migration, regulates actin cytoskeletal dynamics [15–18]. Studies report that the WAVE complex is mostly overexpressed in tumors and has diverse functions during cancer invasion and metastasis [17–19]. The cytoplasmic FMR interacting protein (CYFIP) protein family, including CYFIP1 and CYFIP2, is a component of the WAVE complex [20]. CYFIP1 plays an important role in the WAVE complex as a polymerization activator of actin, but the WAVE complex containing CYFIP2 plays the opposite role. Recent reports revealed that CYFIP2 is a tumor-suppressor involved in chemoresistance and the immune microenvironment in various tumors [21, 22].

In this study, we demonstrated that ELK3 plays a critical role in regulating actin dynamics and filopodia protrusions from the cell membrane of TNBC cells by suppressing expression of CYFIP2, which is a repressor of actin accumulation. Specifically, our findings highlight the significance of the ELK3-CYFIP2 axis as a key regulator driving two major metastatic features of TNBC (enhanced migration and adhesion capacity), thereby facilitating immune evasion from NK cells.

## Materials and methods

### Cell culture

The human TNBC cell lines (MDA-MB-231, Hs578T) and the human NK cell line NK-92MI were obtained from the American Type Culture Collection. ELK3-knockdown (KD) MDA-MB-231 and Hs578T cell lines were previously described [12, 23]. Individual clones with stable suppression of ELK3, achieved through shRNA targeting to the 3’-UTR region (5’-GCCACAATTAAGGAC TCAT-3’), were selected and used in this study. Control cell lines for ELK3KD TNBC cells were established by transducing the cells with a non-silencing shRNA in a matching vector. ELK3 rescue in ELK3KD cells were achieved by transiently transfecting an ELK3-expressing plasmid into the ELK3KD cells. MDA-MB-231, Hs578T, and ELK3KD cells were cultured in Dulbecco’s modified Eagle’s medium (DMEM; Gibco/Life Technologies, GrandIsland, New York, USA). The medium used to culture Hs578T cells was supplemented with 0.01 mg/mL insulin (Sigma-Aldrich, St. Louis, USA). The NK-92MI cell line was cultured in α-minimum essential medium (α-MEM; Gibco/Life Technologies) supplemented with 2 mM L-glutamine (Gibco/Life Technologies), 0.1 mM β-mercaptoethanol (Gibco/Life Technologies), 0.02 mM folic acid (Sigma-Aldrich), and 0.2 mM inositol (Sigma-Aldrich). All media were supplemented with 10% fetal bovine serum and 1% penicillin/streptomycin (Gibco/Life Technologies).

### Plasmid DNA and siRNA

MDA-MB-231 and Hs578T cells were genetically engineered to achieve stable knockdown of ELK3 using retroviral vectors expressing shRNA targeting ELK3, following described in previous studies [23]. The LifeAct-mEGFP-7 expressing plasmid was obtained from Addgene (Massachusetts, USA), and the DNA encoding LifeAct-mEGFP-7 was used to yield a pCDH-LifeAct-mEGFP plasmid. Detailed information regarding the DNA plasmid constructs and siRNA utilized in this study can be found in Supplemental Table 1.

### RNA-sequencing data analysis

To confirm which of the genes encoding each WAVE component shows a negative correlation with ELK3 expression, Gene Expression Omnibus (GEO) (RNA-seq; GSE197575) data deposited at the National Center for Biotechnology Information was used [12].

### Cell migration assay

An 8.0 μm Transwell insert system (Corning, Arizona, USA) was utilized to analyze migration of cancer cells. Briefly, 1 × 10^4^ cells in serum-free DMEM were seeded onto the insert, which was then placed in a 24-well plate containing complete medium. Following a 48 h incubation at 37 °C, the cells that migrated to the lower surface of the insert filter were fixed with 4% paraformaldehyde and stained with crystal violet (Sigma-Aldrich) at room temperature (RT) for 30 min. The number of cells that migrated to the lower chamber were examined under an optical microscope.

### Cell adhesion assay

Briefly, 2.5 × 10^5^ cells were seeded onto a 96-well plate pre-coated with collagen type 1 (Sigma-Aldrich). After incubating the plate at 37 °C for 30 min, non-adherent cells were washed away and the remaining cells were stained with crystal violet (Sigma-Aldrich) at RT for 10 min. Adherent cells were examined under an optical microscope.

### RNA extraction and quantitative RT-PCR

Total RNA was extracted from cancer cells using Trizol (Invitrogen, California, USA) and 1 μg of total RNA was used for cDNA synthesis using the LeGene 1st strand cDNA synthesis system (LeGene Biosciences, California, USA). Quantitative PCR (qPCR) was carried out using TOPreal TM qPCR 2XPreMIX (Enzynomics, Daejeon, Republic of Korea) and the CFX Connect Real-Time PCR Detection System (Bio-Rad Laboratories, California, USA). Expression of mRNA was normalized to that of GAPDH using the comparative cycle method. The primers used in this study are listed in Supplemental Table 2.

### Luciferase assay

A 1.5 kbp fragment of the CYFIP2 promoter region (−1450 bp ~ + 50 bp) was synthesized by Cosmo Genentech (Daejeon, Republic of Korea) and cloned into the pGL3 basic plasmid. ELK3KD MDA-MB-231 cells were transfected with the plasmid using Lipofectamine 2000 (Invitrogen). After 48 h, the transfected cells were harvested and lysed using cell lysis buffer (Cell Signaling Technology, Massachusetts, USA). The cell lysate was analyzed for luciferase activity using the Dual-Luciferase Reporter Assay System (Promega, Wisconsin, USA). The activity of firefly luciferase was normalized to the corresponding values for Renilla luciferase.

### Western blot analysis

Cancer cells were lysed in radioimmunoprecipitation assay (RIPA) buffer (Cell Signaling Technology) supplemented with protease and phosphatase inhibitor cocktail buffer (GenDEPOT, Texas, USA). The concentration of the extracted proteins was determined in a BCA assay. After proteins were denatured by heating at 95 °C for 10 m, 60 µg of protein was separated by sodium dodecyl sulfate polyacrylamide gel electrophoresis (SDS-PAGE) and transferred to a polyvinylidene fluoride membrane (Bio-Rad Laboratories). The membrane was blocked with 4% bovine serum albumin (BSA) and incubated overnight at 4 °C with a primary antibody, followed by a secondary antibody at RT for 1 h. Immunoreactivity was detected using Enhanced Chemiluminescence solution (Thermo Fisher Scientific, Massachusetts, USA) and visualized using ChemiDoc™ XRS + system or ImageQuant LAS 4000 system (GE HealthCare, Barrington, IL, USA). The antibodies used in this study are listed in Supplemental Table 3.

### Chromatin immunoprecipitation (ChIP) assay

Briefly, 1 × 10^6^ ELK3KD MDA-MB-231 cells were seeded in a 100 mm dish. On the next day, the cells were transfected with a Flag-ELK3-expressing plasmid and incubated at 37 °C for 24 h. The cells were then fixed with 1% paraformaldehyde for 15 min to cross-link proteins and genomic DNA. To stop the cross-linking reaction, glycine was added at a final concentration of 125 mM. Cell lysates were prepared using cell lysis buffer (Cell Signaling Technology) supplemented with a protease/phosphatase inhibitor cocktail (Thermo Fisher Scientific). After fragmentation of genomic DNA by sonication, the cell lysates were centrifuged at 15,493 × g for 15 min at 4 °C and the supernatant containing fragmented genomic DNA was collected for immunoprecipitation. The protein concentration and total volume of lysates from all samples were equalized. Immunoprecipitation was performed at 4 °C overnight using protein A/G magnetic beads (Thermo Fisher Scientific) and either an anti-Flag antibody or rabbit IgG. The genomic DNA–protein-antibody complexes were washed sequentially (twice for 10 min each) with 1 × RIPA buffer, 1 × RIPA buffer with 300 mM NaCl (twice for 10 min each), LiCl buffer (twice for 10 min each), and TE buffer (once for 10 min). To separate the DNA–protein complexes, a solution containing proteinase K (Sigma-Aldrich) and 10% SDS was added to TE buffer, and the mixture was incubated at 65 °C overnight. The immunoprecipitated DNA was purified using the phenol/chloroform solution and then used for ChIP-qPCR analysis. The amount of immunoprecipitated chromatin was calculated as a percentage of the input.

### CFSE/7-AAD assay

NK cells were stained for 20 min with 1 µM Cell Trace CFSE (Invitrogen) and then co-cultured for 4 h with cancer cells as target cells. Then, the cells were stained with 7-aminoactinomycin D (7-AAD, Thermo Fisher Scientific) to identify dead cells using a CytoFLEX flow cytometer (Beckman Coulter, Indiana, USA).

### Immunocytochemistry

To visualize actin dynamics, 1 × 10^5^ cells were seeded on coverslips, placed in a 12-well plate, and incubated overnight at 37 °C. The cells were then fixed for 30 min with 4% paraformaldehyde (PFA), followed by permeabilization for 10 min with 0.1% Triton X-100. Afterwards, the cells were pre-incubated for 30 min at RT with 1% BSA blocking solution. Cells were incubated overnight at 4 °C with primary antibodies (diluted 1:500), followed by a secondary antibody (Alexa Fluor 594 or Alexa Fluor 488 phalloidin, both 1:1000; Thermo Fisher Scientific). The stained cells were observed under a confocal microscope, and the length and number of filopodia were quantified using Image J software (ImageJ, Bethesda, MD, USA).

### Time-lapse observation of the actin accumulation of cancer cells in the presence of NK cells

NK-92MI cells, which were stained with a cell trace (Invitrogen) and LifeAct-mEGFP-expressing cancer were seeded into a 96-well confocal plate. The actin movement in cancer cells was assessed under a time-lapse microscope. A modified Olympus FV3000 microscope, fitted with a 40 × (UPlanXApo, NA = 0.98) and a 60 × (UIPlanXApo, NA-1.42) objective lens, and an ANDOR Zyla 4.2 sCOMS camera was used for time-lapse observations. To image living cells, the microscope stage was equipped with an Olympus FV3000 incubation system (Live Cell Instruments, Seoul, Republic of Korea), which maintained the cell cultures at 37 °C, 5% CO_2_. Images acquired during these experiments were analyzed by CellSense software (Olympus, Ishikawa-machi, Hachioji, Tokyo, Japan) and Image J.

### In vivo animal experiments

MDA-MB-231 lung metastasis model were established in 6-week-old female NSG mice (NOD-prkdcscid^em1^Il-2rg^em1^) obtained from JA BIO (Gyeonggi-do, Republic of Korea). Briefly, 1 × 10^6^ MDA-MB-231 cells, engineered to express GFP and luciferase, control, ELK3KD and CYFIP2-silenced ELK3KD cells were injected intravenously. Then, to evaluate in vivo responses to NK cells, 3 × 10^6^ NK-92MI cells were injected after 1 h. The GFP positive cells in the lung was quantified 3 days later using flow cytometry. GFP-labeled tumor cells were visualized by observing frozen lung tissue (sectioned at 8 µm) under a Zeiss LSM510 microscope.

All mice were maintained in a semi-specific pathogen-free animal facility at CHA University (Seongnam, Republic of Korea). Animal experiments were approved by the Institutional Animal Care and Use Committee (IACUC 230135) of CHA University and were carried out in accordance with approved protocols.

### Kaplan–Meier analysis

The probability of survival was calculated using the Kaplan–Meier (KM) Plotter Online Tool (http://www.kmplot.com) to evaluate the relationship between the differential expression of gene and survival in patients with breast cancer [24]. Patients were selected 392 based on ‘negative’ estrogen receptor status, ‘negative’ progesterone receptor status, a ‘negative’ human epidermal growth factor receptor 2. The ratio of genes was calculated as numerator: 215785_s_at (CYFIP2)/ denominator: 206127_at (ELK3). The probability of survival was calculated using the KM method, and log-rank tests were used to calculate the *p*-values.

### Statistical analysis

Statistical analysis was conducted using GraphPad Prism software version 7 (GraphPad Software, San Diego, CA, USA). Data are presented as the mean ± standard deviation (SD), or as the standard error of the mean (SEM). Statistical significance was defined as follows: *P < 0.05, ** P < 0.01, *** P < 0.001, or **** P < 0.0001. The term “NS” denotes non-significant results.

## Results

### ELK3 expression is associated with protrusion of filopodia from the TNBC cells

**Fig. 1:**
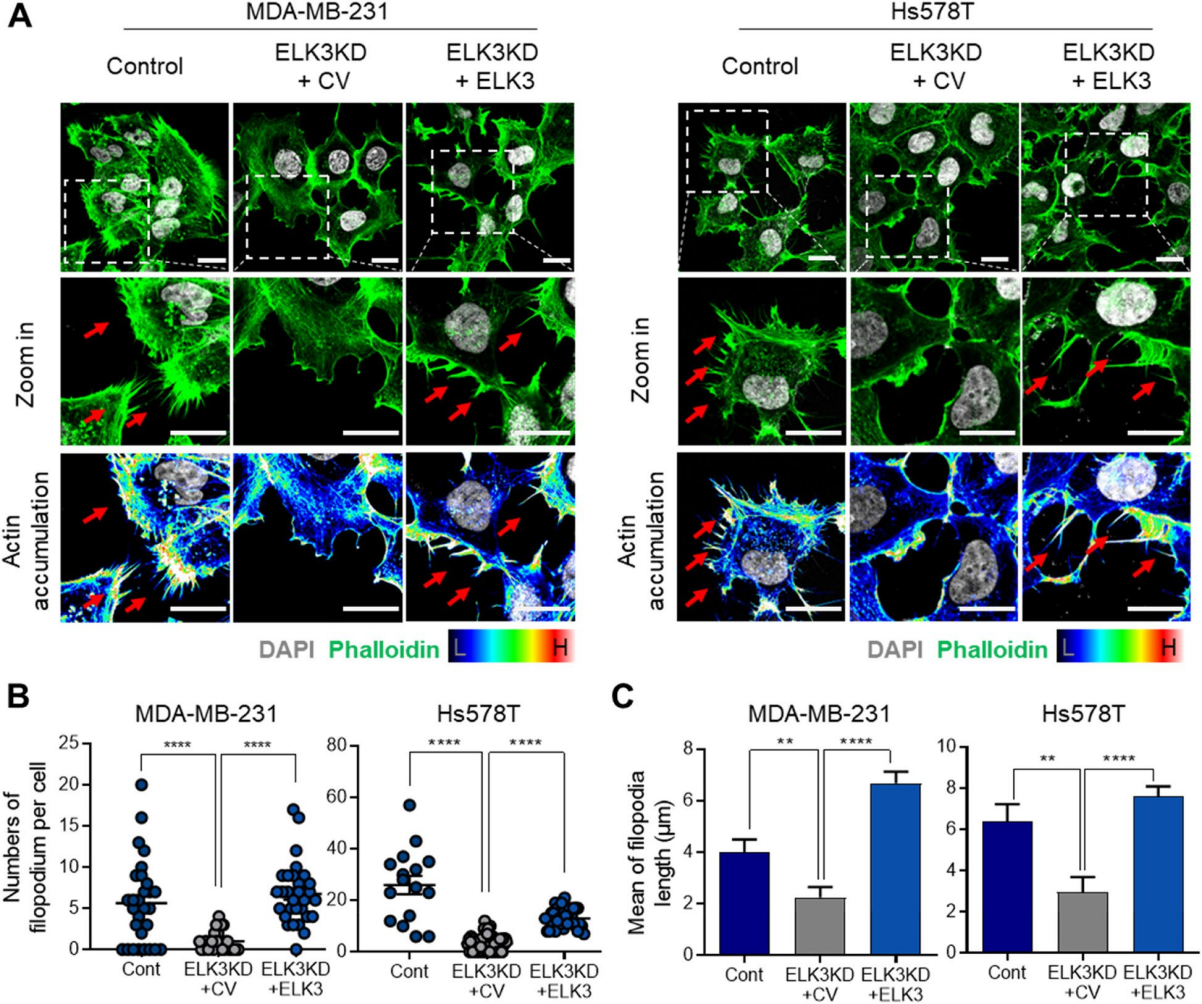
ELK3 expression levels are associated with filopodia protrusion in TNBC cells. **A** Protrusion of filopodia from MDA-MB-231 and Hs578T cells was compared with that from ELK3KD cells transfected with an empty matching plasmid (control vector; CV) or an ELK3-expressing vector (ELK3). Cells were stained with DAPI and phalloidin. Actin accumulation associated with filopodia formation was visualized using fluorescence microscopy; representative protrusions are indicated by red arrows. Scale bar, 20 µm. **B** The number of filopodia per cell were quantified and is presented as individual dots. (MDA-MB-231 cells, *n* = 29, 30, and 30, respectively, Hs578T cells, *n* = 16, 67, and 35, respectively.) **C** The length of filopodia are presented in a graph. (MDA-MB-231 cells, *n* = 29, 30, and 30, respectively, Hs578T cells, *n* = 14, 17, and 17, respectively.) Data are presented as the standard error of the mean (SEM). Control (Cont) = sh control of MDA-MB-231 or Hs578T cells; ELK3KD = ELK3KD of MDA-MB-231 or Hs578T cells. *P < 0.05; **P < 0.01; ***P < 0.001; ****P < *0.0001*

Filopodia are slender, finger-like protrusions that extend from the cell surface and play a critical role in cancer cell motility, migration, and metastasis [25]. In line with this, highly metastatic MDA-MB-231 cells exhibit numerous structures resembling filopodia in vitro [26]. Given that ELK3KD MDA-MB-231 cells lose their metastatic properties [23], we questioned whether ELK3KD cells have fewer filopodia. To confirm this, we rescued ELK3 expression in ELK3KD of MDA-MB-231 or Hs578T cells transiently. As expected, staining with phalloidin to visualize actin filaments revealed that ELK3KD cells exhibited fewer cell surface protrusions than control cells; however, upon restoration of ELK3 expression, the cell surface showed more protrusions (Fig. 1A, left). A similar pattern was observed in Hs578T cells, another TNBC cell line (Fig. 1A, right). Quantification in Fig. 1A indicates a significant correlation between ELK3 expression and both the number and mean length of filopodia per MDA-MB-231 and Hs578T cell (Fig. 1B–C).

### ELK3 functions as a transcriptional repressor of CYFIP2 in TNBCs

Filopodia comprises the WAVE complex (i.e., WASF also known as WAVE), NCKAP1, ABI, and BRICK1 (or their homologs), and CYFIP, which connects the membrane to the cytoskeleton [18, 27, 28]. Since ELK3 functions as a transcriptional repressor in TNBCs [12, 29], we assessed which of the genes encoding each WAVE component shows a negative correlation with ELK3 expression by analyzing RNA-sequencing data from MDA-MB-231 cells, ELK3KD cells, and ELK3-rescued ELK3KD cells.

**Fig. 2:**
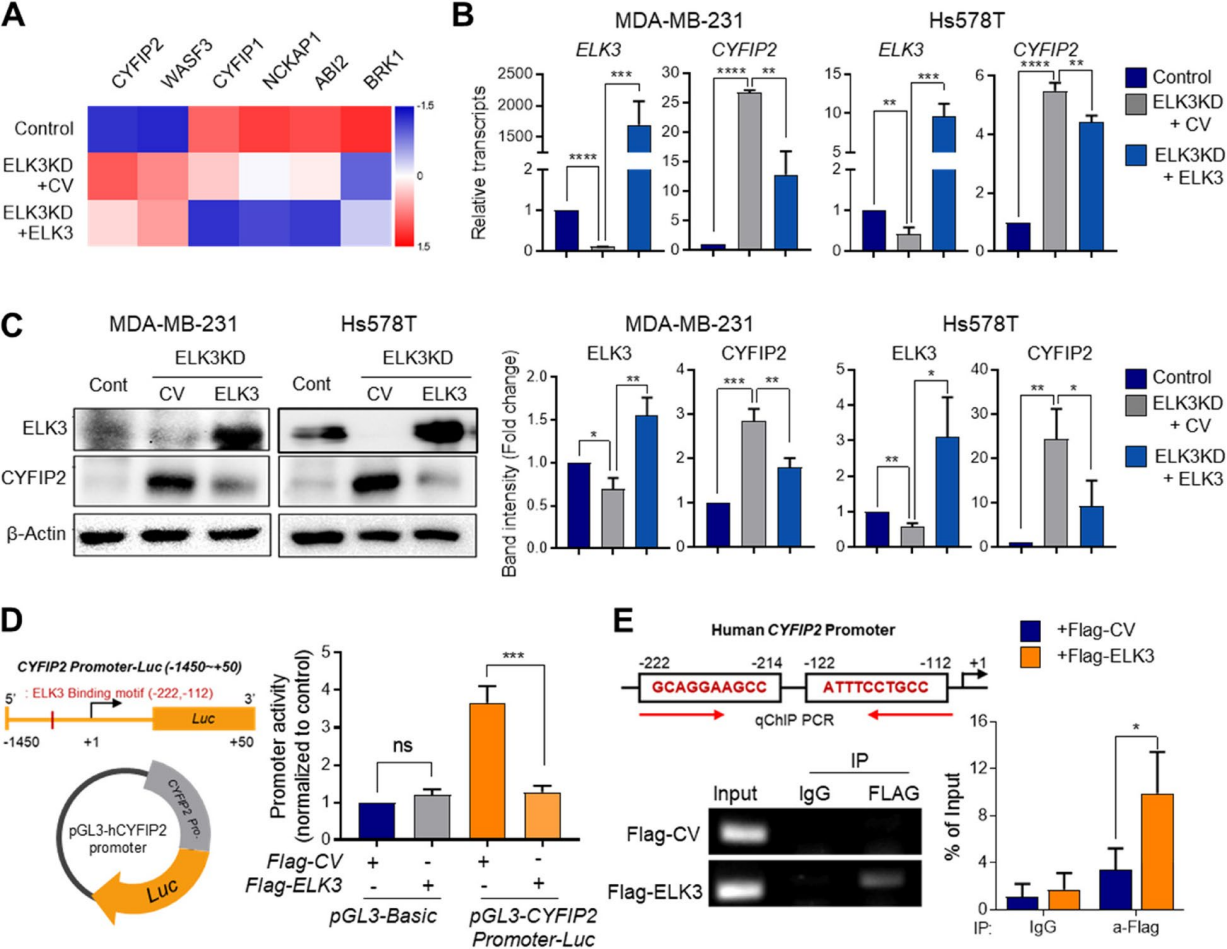
ELK3 transcriptionally represses expression of *CYFIP2* in TNBCs. **A** Heat map displaying the expression levels of WAVE complex subunits (CYFIP2, WASF3, BRK1, CYFIP1, NCKAP1, ABI2) identified by RNA-sequencing analysis of MDA-MB-231 control, ELK3KD cells and ELK3KD + ELK3 rescued cell. **B**–**C** Quantitative RT-PCR and immunoblot analysis of CYFIP2 in control and ELK3KD TNBC cells that were transfected with a control vector (CV) or an ELK3-expressing vector (ELK3). Control (Cont) = sh control of MDA-MB-231 or Hs578T cells; ELK3KD = ELK3KD of MDA-MB-231 or Hs578T cells. **D** Luciferase reporter assay showing activity of the *CYFIP2* promoter. ELK3KD MDA-MB-231 cells were transfected with the indicated plasmid combinations for 48 h, followed by a luciferase assay. **E** ChIP-qPCR analysis of ELK3 binding to the *CYFIP2* promoter. A Flag-ELK3-expressing plasmid or Flag-control plasmid was transfected into ELK3KD cells for 48 h, and Flag-immunoprecipitates were subjected to qPCR using primers specific for the *CYFIP2* promoter region (−1450 to + 50 bp). All data were derived from at least three independent biological experiments. Data are presented as the mean ± standard deviation (SD). NS indicates no statistical significance. *P < 0.05; **P < 0.01; ***P < 0.001; ****P < *0.0001*

Although CYFIP2 and WASF3 expressions were repressed in ELK3KD cells, rescuing ELK3 expression in ELK3KD cells did not significantly suppressed WASF3/WAVE3 expression. This suggests that ELK3 may not directly regulate WASF3/WAVE3 in the same manner it regulates CYFIP2. Based on this, we focused on CYFIP2 in our study because it demonstrated a clear negative correlation with ELK3 expression in the RNA-sequencing data (Fig. 2A). CYFIP2, a negative regulator of actin polymerization, inhibits filopodia formation [20]. Consistent with a results shown in Figure 2A, quantitative analysis demonstrated that *CYFIP2* mRNA and protein were upregulated in ELK3KD of MDA-MB-231 and Hs578T cells, while their expression decreased upon restoration of ELK3 (Fig. 2B, C). We identified an ELK3 binding motif on the promoter of *CYFIP2* near to the transcription initiation site across human, murine, and rat species (Supplemental Figure 1). To prove that *CYFIP2* is a direct downstream target of ELK3 in TNBCs, we employed a luciferase reporter assay using a construct encoding luciferase harboring bp −1450 to +50 of the *CYFIP2* promoter region. As shown in Figure 2D, ELK3 significantly inhibited *CYFIP2* promoter activity. Direct binding of ELK3 to the *CYFIP2* promoter was confirmed via a ChIP assay showing Flag-ELK3 bound to the corresponding motif (Fig. 2E). These findings suggest that ELK3 functions as a transcriptional repressor of *CYFIP2* in TNBCs.

### The ELK3-CYFIP2 axis regulates the metastatic characteristics of TNBCs by modulating filopodia protrusion

**Fig. 3:**
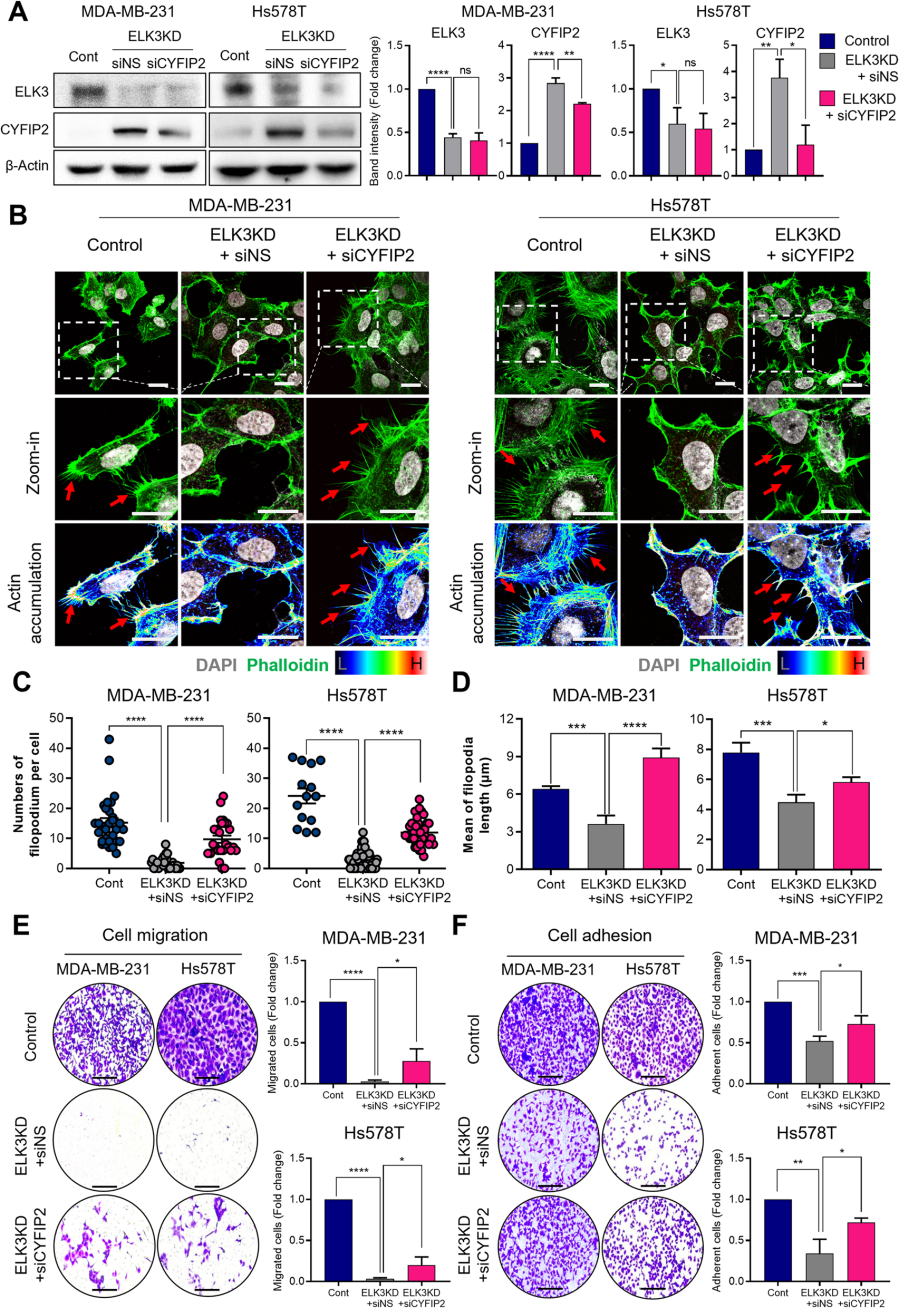
ELK3-CYFIP2 axis regulates metastatic nature of TNBCs by modulating filopodia protrusion. **A** Immunoblot analysis confirms the activity of siRNA targeting CYFIP2 (siCYFIP2) in ELK3KD MDA-MB-231 and Hs578T cells. ELK3KD TNBC cells transfected with a non-specific siRNA (siNS) or siCYFIP2. Relative band intensity of ELK3 and CYFIP2. Data are presented as the mean ± SD. **B** Filopodia formation was observed after staining with DAPI and phalloidin. Actin accumulation of filopodia formation was visualized using fluorescence microscopy; representative protrusions are indicated by red arrows. Scale bar, 20 µm. **C** The number of filopodia per cell were quantified, and is presented as individual dots. (MDA-MB-231 cells, *n* = 30, 30, and 26, respectively, Hs578T cells, *n* = 14, 30, and 30, respectively.) **D** The length of filopodia are presented in a graph. (MDA-MB-231 cells, *n* = 30, 30, and 26, respectively, Hs578T cells, *n* = 16, 30, and 30, respectively.) Data are presented as the SEM. **E**–**F** Representative images showing migration and adhesion of the indicated cells. Scale bar, 200 µm. All data were derived from at least three independent biological experiments. Data are presented as the mean ± SD. Control (Cont) = sh control of MDA-MB-231 or Hs578T cells; ELK3KD = ELK3KD of MDA-MB-231 or Hs578T cells. NS indicates no statistical significance. *P < 0.05; **P < 0.01; ***P < 0.001; ****P < *0.0001*

To further explore the biological function of the ELK3-CYFIP2 axis in TNBCs, we assessed the impact of *CYFIP2* suppression on the metastatic traits of ELK3KD TNBCs, which had lost their metastatic characteristics due to ELK3KD. Successful CYFIP2 suppression was accomplished through transfection of siRNA into ELK3KD MDA-MB-231 and Hs578T cells (Fig. 3A, Supplemental Figure 2). Notably, CYFIP2 suppression resulted in a resurgence of cell surface protrusions in both ELK3KD MDA-MB-231 and Hs578T cells, as evidenced by the increase in the average number of filopodia per cell and the average length (Fig. 3B–D). To further confirm the association of actin protrusions with CYFIP2, we utilized lysophosphatidic acid (LPA), which is known to induce Arp2/3 activation. We observed actin accumulation at the cell edges through F-actin staining after LPA treatment. As shown in the arrow, we confirmed that filopodium formation increased with LPA treatment. This was more prominent in ELK3KD (Supplemental Figure 3A–C). Also, we analyzed activated Rac1/Cdc42 and RhoA activation, its downstream effector ROCK activity, via western blot after LPA stimulation. Upon LPA treatment, RhoA and Rac1/Cdc42, which are the basis for activation of GTPases, were upregulated, as was the case when Arp2/3 was activated (Supplemental Figure 3D).

Additionally, to investigate the function of ELK3-CYFIP2 axis in EMT, we performed experiments to analyze the expression of classical EMT markers at the RNA and protein level (Supplemental Figure 4). These results suggest that while ELK3 partially acts as a regulator of EMT, CYFIP2 may influence cell migration more directly through its role in actin polymerization rather than through direct modulation of EMT markers.

Furthermore, we found that suppressing CYFIP2 in ELK3KD of MDA-MB-231 and Hs578T cells enhanced their migration ability, which had been impaired by ELK3 suppression (Fig. 3E). Additionally, the low cell adhesion observed in ELK3KD cells increased when CYFIP2 was suppressed (Fig. 3F). These results indicate that the ELK3-CYFIP2 axis regulates key metastatic properties in TNBCs, including filopodia formation, migration, and adhesion.

### The ELK3-CYFIP2 axis affects the sensitivity of TNBCs to NK cells by regulating actin accumulation

**Fig. 4:**
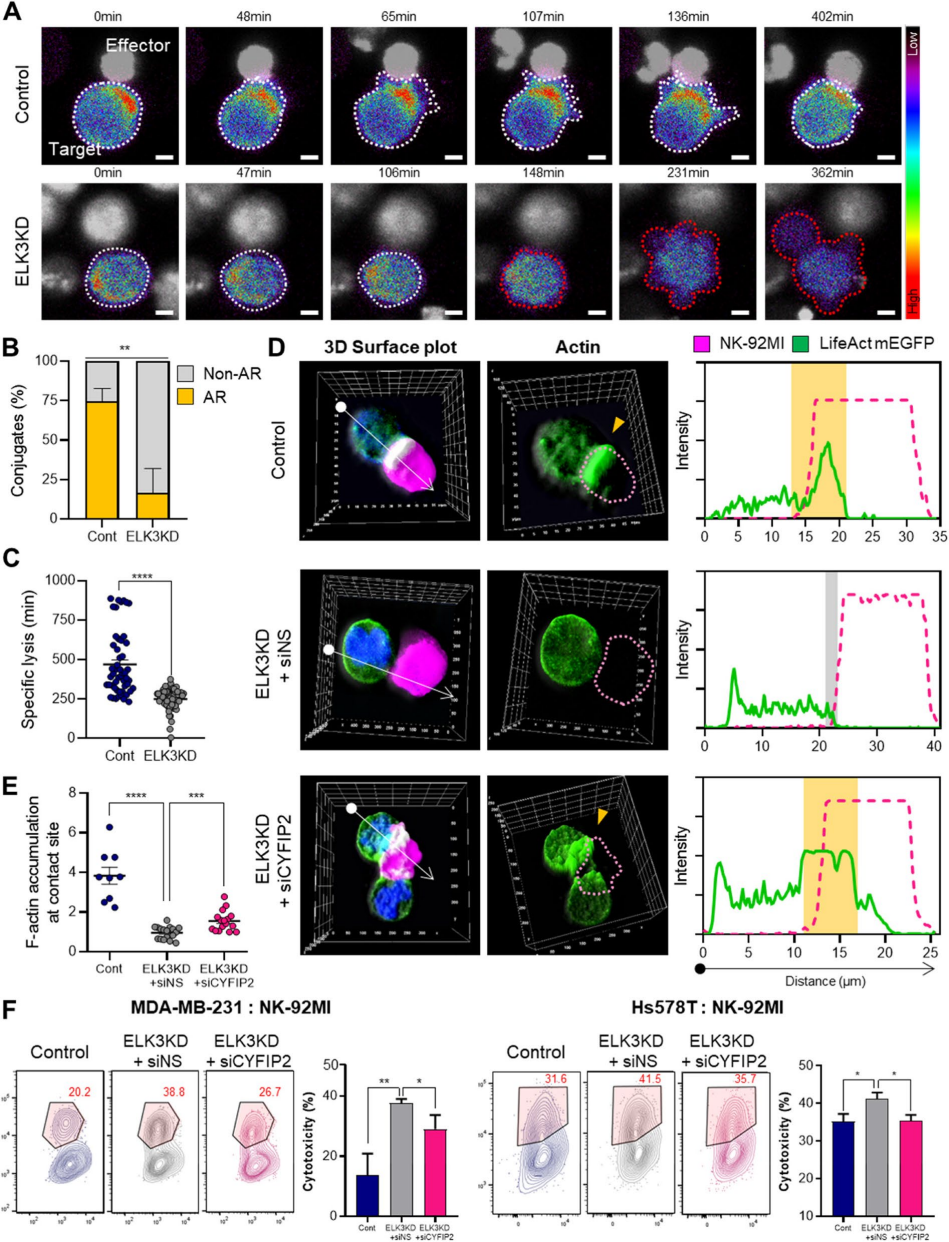
The ELK3-CYFIP2 axis regulates immune sensitivity of TNBCs to NK cells by regulating actin accumulation. **A** Time-lapse images showing the process of filopodia protrusion and actin accumulation in MDA-MB-231 control or ELK3KD cells at the region of contact with NK-92MI cells (E:T ratio = 1:1). The cancer cells were engineered to express LifeAct-mEGFP, which enables visualization of actin. Scale bar, 5 µm. **B** Quantification of MDA-MB-231 control or ELK3KD cells showing actin accumulation at the contact site with NK-92MI cells. Cancer cells showing an actin response are denoted as AR (in yellow), and cancer cells with no actin response are denoted as Non-AR (in gray). **C** The time taken to lyse of MDA-MB-231 control or ELK3KD cells after contact with NK-92MI. Data are presented as individual dots. (Cont: *n* = 51, and ELK3KD: *n* = 57.) **D** Actin accumulation at the contact site between MDA-MB-231 control cells, ELK3KD cells, ELK3KD cells transfected with siCYFIP2, and NK-92MI cells was observed under a fluorescence microscope. **E** Actin intensity at the contact site with NK-92MI cells was quantified. (Cont: *n* = 9, ELK3KD + siNS: *n* = 17 and ELK3KD + siCYFIP2: *n* = 16.) Data are presented as the standard error of the mean (SEM). **F** Immune response of MDA-MB-231 and Hs578T control cells, and ELK3KD cells, transfected with non-specific siRNA (siNS) or siCYFIP2 to NK-92MI. Cytotoxic activity of NK-92MI against cancer cells was measured in a CFSE/7-AAD assay (E:T ratio = 10:1). Control (Cont) = sh control of MDA-MB-231 or Hs578T cells; ELK3KD = ELK3KD of MDA-MB-231 or Hs578T cells. The experiments were performed in triplicate. Data are presented as the mean ± SD. *P < 0.05; **P < 0.01; ***P < 0.001; ****P < 0.0001

Filopodia formation, an important factor in metastatic nature, is driven by dynamic remodeling of actin, and a recent report revealed that this actin accumulation in breast cancer cells induces resistance to NK cell-mediated cytotoxicity [13]. Visualization of GFP-fused actin in MDA-MB-231 control and ELK3KD cells co-cultured with NK-92MI cells revealed protruded actin accumulation at the contact site in control cells, contrasting with ELK3KD cells (Fig. 4A, Supplemental Figure 5). Quantification of cancer cells displaying GFP-fused actin accumulation at the site of contact with NK cells further supported the observation that ELK3KD cells exhibit reduced actin accumulation following contact with NK cells (Fig. 4B). Also, ELK3KD cells exhibit a significantly shorter killing time by NK cells (Fig. 4C), indicating that actin response influences cytotoxicity. To confirm whether the ELK3-CYFIP2 axis in TNBCs is involved in actin accumulation during the immune response against NK cells, we transfected siCYFIP2 into ELK3KD TNBCs. Suppression of CYFIP2 increases actin accumulation in ELK3KD cells upon contact with NK cells (Fig. 4D–E, Supplemental Figure 6). Consistently, Fig. 4F shows significantly reduced NK cytotoxicity in ELK3KD-TNBCs transfected with siCYFIP2. These results highlight the role of the ELK3-CYFIP2 axis in the immune sensitivity of these cancer cells to NK cells through actin accumulation.

### The effect of pharmacological inhibition of actin remodeling on the immune response of MDA-MB-231 cells to NK cells

Our data suggest that actin assembly at the contact site between ELK3KD cells and NK cells was impaired by upregulated expression of CYFIP2, thereby increasing their sensitivity to NK cells. Based on the report that CYFIP2 induces destabilization of WAVE complexes [20], we hypothesized that Arp2/3, which acts downstream of WAVE, is impaired in ELK3KD TNBCs. To further confirm this, we investigated whether ELK3KD cells respond to a chemical inhibitor of actin remodeling in the context of immune sensitivity to NK cells.

**Fig. 5:**
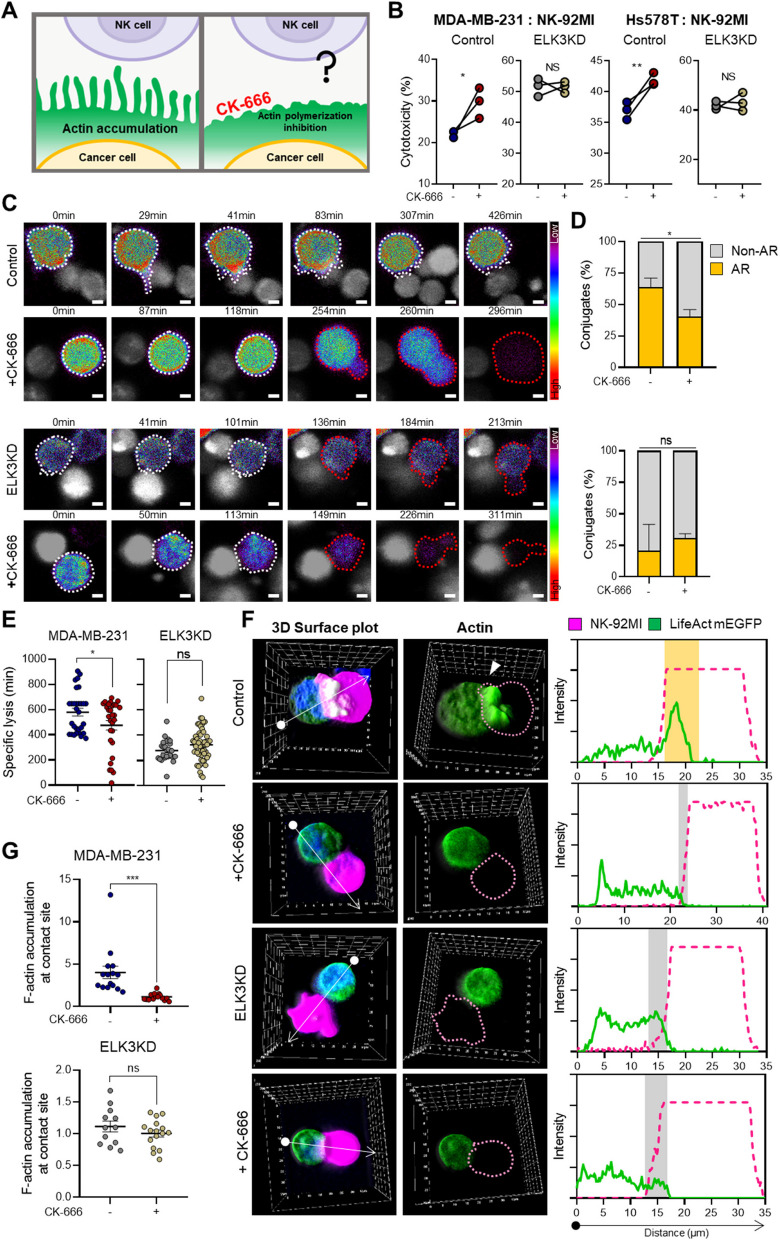
Chemical inhibition of actin remodeling sensitizes immune response of MDA-MB-231 cells, but not ELK3KD cells, to NK cells.** A** Schematic showing the hypothesis underlying the immune response of CK-666 to inhibit actin polymerization. **B** Immune response of control and ELK3KD TNBC (MDA-MB-231 and Hs578T) in the presence or absence of CK-666. The cytotoxic activity of NK-92MI against cancer cells was measured in a CFSE/7-AAD assay (E:T ratio = 10:1). The experiments were performed in triplicate. Data are presented as the mean ± SD. **C** Time-lapse images showing filopodia protrusion and actin accumulation in MDA-MB-231 control or ELK3KD cells at the region of contact with NK-92MI cells (E:T ratio = 1:1) in the presence or absence of CK-666. Cancer cells were engineered to express LifeAct-mEGFP, which enables visualization of actin. Red dotted lines denote cancer cells undergoing lysis after contact with NK-92MI cells. Scale bar, 5 µm. **D** Quantification of MDA-MB-231 control or ELK3KD cells showing actin accumulation at the contact site with NK-92MI cells in the presence or absence of CK-666. Cancer cells showing an AR are denoted in yellow, and those showing Non-AR in gray. **E** Time taken for lysis of MDA-MB-231 control or ELK3KD cells after contact with NK-92MI in the presence or absence of CK-666. (MDA-MB-231 cells, -: *n* = 30, + : *n* = 29 and ELK3KD, -: *n* = 25, + : *n* = 56 respectively.) **F** Actin accumulation at the site at which MDA-MB-231 control and ELK3KD cells contact NK-92MI cells was assessed in the presence or absence of CK-666 and observed under a fluorescence microscope. **G** Actin intensity at the contact site between MDA-MB-231 control and ELK3KD cells and NK-92MI cells in the presence or absence of CK-666. (MDA-MB-231 cells, -: *n* = 15, + : *n* = 15 and ELK3KD, -: *n* = 12, + : *n* = 15 respectively.) Control (Cont) = sh control of MDA-MB-231 or Hs578T cells; ELK3KD = ELK3KD of MDA-MB-231 or Hs578T cells. Data are presented as the SEM. *P < 0.05; **P < 0.01; ***P < 0.001

As expected, CK-666, which disrupts actin cytoskeleton dynamics and actin remodeling by inhibiting Arp2/3 (Fig. 5A) [30], had no impact on ELK3KD MDA-MB-231 and Hs578T cell immune responses to NK cells, but significantly enhanced the sensitivity of control cells (Fig. 5B). Furthermore, we assessed changes in cancer cell susceptibility to NK cell-mediated cytotoxicity by co-culturing LPA-treated cancer cells with NK cells (Supplemental Figure 7). These results show that while LPA treatment does not significantly affect NK cell sensitivity in control, it significantly decreases NK cell-mediated cytotoxicity in ELK3KD cells. This indicates that the ELK3-CYFIP2 axis may influence the sensitivity of cancer cells to NK cell-mediated cytotoxicity, particularly in the context of Arp2/3 activation through LPA. Time-lapse microscopy revealed CK-666 inhibited filopodia protrusion in control cells at the NK cell contact site, whereas ELK3KD MDA-MB-231 cells did not respond to this chemical (Fig. 5C, Supplemental Figure 8). Quantification confirmed that CK-666 had no impact on ELK3KD cell actin responses to NK cell contact (Fig. 5D). Consistent with its impact on actin accumulation in MDA-MB-231 and ELK3KD cells, CK-666 reduced the time taken for NK cells to kill MDA-MB-231 control cells, but did not affect the killing time of ELK3KD cells (Fig. 5E). Next, we assessed the impact of CK-666 on relative actin accumulation at the contact site between cancer cells and NK-92MI cells. While MDA-MB-231 cells showed reduced GFP-fused actin accumulation upon CK-666 treatment, there was no significant change in ELK3KD cells (Fig. 5F–G). These results indicate that Arp2/3 is already inactive in ELK3KD of TNBCs, leading to diminished actin accumulation and enhanced susceptibility to NK cells.

### The ELK3-CYFIP2 axis plays a role in the response of TNBC to NK cell immunosurveillance and in survival of patients with TNBC

**Fig. 6:**
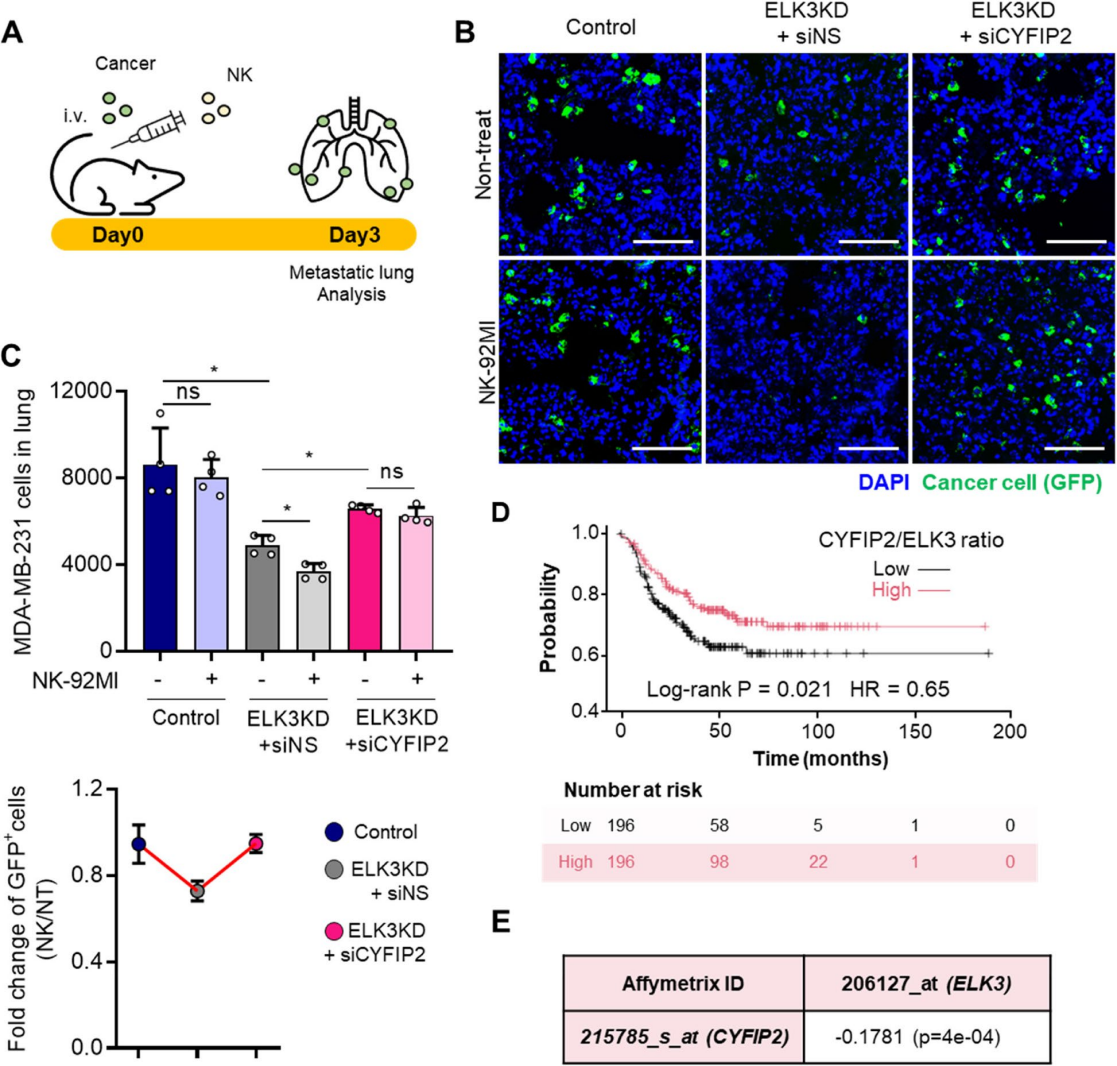
ELK3-CYFIP2 axis-mediated regulation of metastasis and NK responses in a mouse model bearing MDA-MB-231 tumors, and its clinical significance in TNBC patients. **A** Schematic of the in vivo experiment: GFP-expressing control, ELK3KD and CYFIP2-silenced ELK3KD MDA-MB-231 cells were injected intravenously into NSG mice. NK cells were injected intravenously 1 h later (each group *n* = 4). **B** Fluorescence images of GFP, indicating extravasated tumor cells in the lungs of mice at 3 days. Nuclei was stained with DAPI. Scale bar, 100 μm. **C** GFP positive cells in the lungs from each group of mice were quantified by flow cytometry. Control (Cont) = sh control of MDA-MB-231 ELK3KD = ELK3KD of MDA-MB-231 cells. Data represents the mean ± SD. *P < 0.05; **P < 0.01; ***P < 0.001. **D** Kaplan–Meier survival analysis of TNBC patients (*n* = 392), who were stratified into ‘high’ and ‘low’ groups based on the CYFIP2/ELK3 ratio. **E** Correlation between ELK3 and CYFIP2 expression levels in 392 patients with TNBC

To determine whether the ELK3-CYFIP2 axis regulates the metastatic ability of TNBC, as well as the NK response in vivo, we performed an extravasation analysis in mice injected with GFP-expressing control cells, ELK3KD cells, or CYFIP2-silenced ELK3KD MDA-MB-231 cells. A total of 1 × 10^6^ cancer cells were injected intravenously into immunodeficient NSG mice, and 3 × 10^6^ NK-92MI cells were injected 1 h later (Fig. 6A). Then, the presence of GFP positive cells in the lung was examined 3 days later. A presence of GFP positive cancer cells was observed in frozen sections of lung tissue from each group of mice (Fig. 6B). Consistent with the in vitro results, compared with the control MDA-MB-231 cells, fewer GFP positive cells were detected in mice injected with ELK3KD cells, whereas the CYFIP2-silenced ELK3KD cells were detected at similar levels to control cells. Notably, when NK-92MI cells were injected in mice already injected with either the control cells or CYFIP2-silenced ELK3KD cells, the number of GFP positive cells remained unchanged; however, the number fell significantly in mice injected with ELK3KD cells. We quantified GFP positive cells in the lung by flow cytometry (Fig. 6C). A consistent presence of GFP positive cancer cells was observed of lung tissue from each group of mice, indicating that the ELK3-CYFIP2 axis influences the response of MDA-MB-231 cells to the anti-cancer activity of NK cells in vivo.

To further validate these findings, we examined the effect of the ELK3-CYFIP2 axis on cancer cell survival in immune-competent mice, both in the presence and absence of NK cells. Using Elk3 knockout 4T1 mouse TNBC cell line (Elk3KO 4T1) (Supplemental Figure 9A), we first confirmed that mouse Elk3 represses Cyfip2 expression. Consistent with our findings in human cells, we observed increased Cyfip2 transcript levels in Elk3KO 4T1 (Supplemental Figure 9B). We then investigated the effect of NK cell-mediated cancer cell clearance using an in vivo mouse tumor model. Endogenous NK cells in Balb/c mice were first depleted by administering anti-Asialo GM1 antibody one day before cancer cell injection, followed by intravenous injection of CFSE-labeled 4T1 cells and subsequent analysis of lung metastasis (Supplemental Figure 9C). Fluorescence microscopy of lung sections and quantitative analysis of remaining cancer cells showed that NK cell depletion significantly affected cancer cell survival in the lungs (Supplemental Figure 9D-E). In the presence of endogenous NK cells (DPBS treatment), Elk3KO 4T1 showed reduced presence in the lungs compared to control cells, indicating enhanced NK cell-mediated clearance of Elk3KO 4T1. However, following NK cell depletion, this difference was abolished, and Elk3KO 4T1 exhibited similar levels to control cells. When Cyfip2 was knocked down in Elk3KO 4T1, the enhanced NK cell-mediated clearance was reversed, suggesting that the increased susceptibility of Elk3KO 4T1 to NK cell-mediated killing is Cyfip2-dependent.

Finally, we used the KM Plotter Online Tool to examine the clinical significance of ELK3 and CYFIP2 expressions in TNBC cell lines and patient. Patients with TNBC (*n* = 392) were classified into ‘high’ and ‘low’ groups based on the CYFIP2/ELK3 ratio. Kaplan–Meier plots demonstrated that patients ‘low’ had significantly shorter survival than patients ‘high’ (Fig. 6D). Moreover, the expression of ELK3 showed a weak but statistically significant negative correlation with that of CYFIP2 (Fig. 6E). Taken together, these findings suggest that the ELK3-CYFIP2 axis has biological activity associated with survival of TNBC patients.

## Discussion

TNBC, an aggressive form of breast cancer with a poor prognosis, lacks estrogen and progesterone receptors, as well as the HER2 protein, rendering both hormone therapy and HER2-targeting drugs ineffective. While surgery and chemotherapy are more effective at the early stages, they are less so at advanced stages, necessitating identification of novel therapeutic targets for TNBC patients. In this study, we identified a pivotal role for ELK3 as a regulator of actin dynamics in TNBC. ELK3 achieves this by inhibiting the expression of CYFIP2, a crucial component of the WAVE complex responsible for polymerizing branched actin networks.

CYFIP1 and CYFIP2 are integral components of the canonical WAVE regulatory complex. Specifically, CYFIP1 initiates actin nucleation, a process crucial for the precise spatiotemporal regulation of actin dynamics [27, 28]. Though CYFIP2 exhibits a high level of similarity to CYFIP1, its biological significance is ambiguous because CYFIP2 expression can be either a protective prognostic factor or a risk factor depending on the specific cancer type [31–33]. Figure 6E suggests that CYFIP2 is a protective prognostic factor in ELK3^low^ TNBC patients.

EMT in cancer cells, a process linked to metastasis, involves cytoskeletal remodeling, which facilitates morphological adaptations and acquisition of migratory and invasive properties. EMT-associated cytoskeletal changes involve extensive remodeling of the cytoskeleton to enable morphological and functional adaptations, including acquisition of migratory and invasive properties [34].

Consequently, these cytoskeletal changes associated with EMT likely enhance the ability of tumor cells to rapidly remodel actin in response to attack by immune cells. Studies report that EMT facilitates escape of tumor cells from cytotoxic immune cells [35, 36]. Supporting this notion, our data reveal that control TNBC cells exhibit a significantly higher capacity than ELK3KD TNBC cells (which have impaired metastatic features) to generate an actin response at the contact site with NK cells. Successful dissemination of cancer cells from the primary tumor site, coupled with evasion of immune cells as they circulate, are critical steps in metastasis to other organs. Overcoming escape of tumor cells from cytotoxic immune cells presents a significant challenge to developing effective immunotherapies for metastatic cancers. Considering the pivotal role of the actin cytoskeleton in remodeling and governance of cancer cell mobility and resistance to immune cell-mediated cell death, targeting the molecular mechanisms involved in regulating actin dynamics holds promise as a therapeutic strategy to address both EMT-mediated metastasis and immune evasion by disseminated cells.

In this context, an intriguing finding from our study is the therapeutic potential of targeting the ELK3-CYFIP2 axis to combat TNBC metastasis and increase the efficacy of immunotherapeutic strategies. As shown in Fig. 6E, TNBC patients with low ELK3 expression and high CYFIP2 expression showed higher survival rates than those with high ELK3 expression and low CYFIP2 expression. By inhibiting ELK3, it may be possible to reduce the invasive capacity of TNBC cells, making them more vulnerable to immune surveillance and cytotoxicity mediated by NK cells. Another crucial aspect discussed in this study is the pleiotropic activity of ELK3 in relation to cancer metastasis and immunosensitivity. ELK3 exhibits a wide range of functions and plays significant roles in these processes. In terms of regulating metastasis, ELK3 is associated with inhibition of specific genes and microRNAs, expression of which shows an inverse correlation with cancer metastasis [37–39]. ELK3 is also involved in other important oncogenic processes such as development of peritumoral lymphatic vessels, which is critical for tumor dissemination. By regulating angiogenic factors like VEGFC, ELK3 promotes growth of lymphatic vessels, thereby facilitating cancer metastasis [40]. Regarding regulation of the immune responses or chemoresistance of TNBCs, studies report that ELK3 renders cancer cells resistant to NK cells and cisplatin (CDDP) chemotherapy by regulating mitochondrial dynamics [12, 29, 41]. For instance, ELK3 directly suppresses genes involved in mitochondrial fission, such as *Drp1* and *Mid51*, and loss of ELK3 expression leads to induction of mitochondrial fission, which is associated with vulnerability of these cells to NK cells, as well as increased chemosensitivity to CDDP [12, 29]. Additionally, ELK3 suppresses chemotactic cytokines such as CXCL16 to inhibit tumor-infiltrating NK cells [41].

In this context, the conceptual novelty of the current investigation lies in the first demonstration of ELK3’s role in suppressing a gene associated with actin cytoskeleton organization, which is a critical determinant of the mechanical integrity of cancer cells. The mechanical integrity of TNBC cells is directly associated with their metastatic potential and their response to immune cell-mediated clearance.

By integrating these novel findings with previously reported data, this study positions ELK3 as a master regulator in TNBC. ELK3 orchestrates metastasis and immune evasion by modulating the expression of numerous genes involved in diverse cellular processes, including EMT, mitochondrial dynamics, and cytoskeletal organization. These findings suggest that metastatic potential and immune evasion in TNBC are interlinked phenotypes, arising as cumulative outcomes of coordinated biological processes regulated by ELK3.

## Conclusion

Our research strongly supports the notion that the ELK3-CYFIP2 axis acts as a actin remodeling regulator that drives metastasis of TNBC, influencing both cancer cell migration and immune sensitivity to NK cells. Overall, our findings suggest that targeting the ELK3-CYFIP2 axis holds promise as a therapeutic strategy for combating metastatic TNBCs. Specifically, inhibiting this axis could hinder cancer cell migration and adhesion while at the same time increasing immune sensitivity to NK cells. Disrupting this pathway is expected to pave the way for more effective treatment strategies for TNBC patients.

## Supplementary Information

Supplementary Material 1.

## Data Availability

All data reported in this paper will be shared by the lead contact upon request.

## Abbreviations

ELK3: E26 transformation-specific transcription factor
CYFIP2: Cytoplasmic-FMR1 interacting protein 2
WASP: Wiskott-Aldrich syndrome protein
WAVE: WASP family verprolin homologous protein
TNBC: Triple-negative breast cancer
NK cell: Natural killer cell
EMT: Epithelial-mesenchymal transition
E:T: Effector:target
7-AAD: 7-Aminoactinomycin D
ELK3KD: Stable knockdown (KD) of ELK3 with shRNA in TNBC cell line
