# Correction: ELK3-CYFIP2 axis-mediated actin remodeling modulates metastasis and natural killer cell responses in triple-negative breast cancer

**DOI:** 10.1186/s13046-025-03388-6

**Published:** 2025-04-16

**Authors:** Seung Hee Choi, Hye Jung Jang, Joo Dong Park, Ki Seo Ryu, Eunchong Maeng, Seohyun Cho, Hail Park, Hae-Yun Jung, Kyung-Soon Park

**Affiliations:** 1 Department of Biomedical Science, CHA University, Seongnam, Republic of Korea; 2 Department of Integrative Biotechnology, Sungkyunkwan University, Suwon, Republic of Korea; 3 Division of Radiation Biomedical Research, Korea Institute of Radiological and Medical Sciences, Seoul, Republic of Korea

**Correction: J Exp Clin Cancer Res 44, 48 (2025)**

10.1186/s13046-025-03309-7

Following the publication of the original article [1], the authors identified an error in Fig. 3 where the images of Fig. 3e and 3f inadvertently overlapped and were not properly replaced as intended in the published version of our manuscript.

The correct figure is presented below:

## Incorrect Fig. 3

**Fig. 3:**
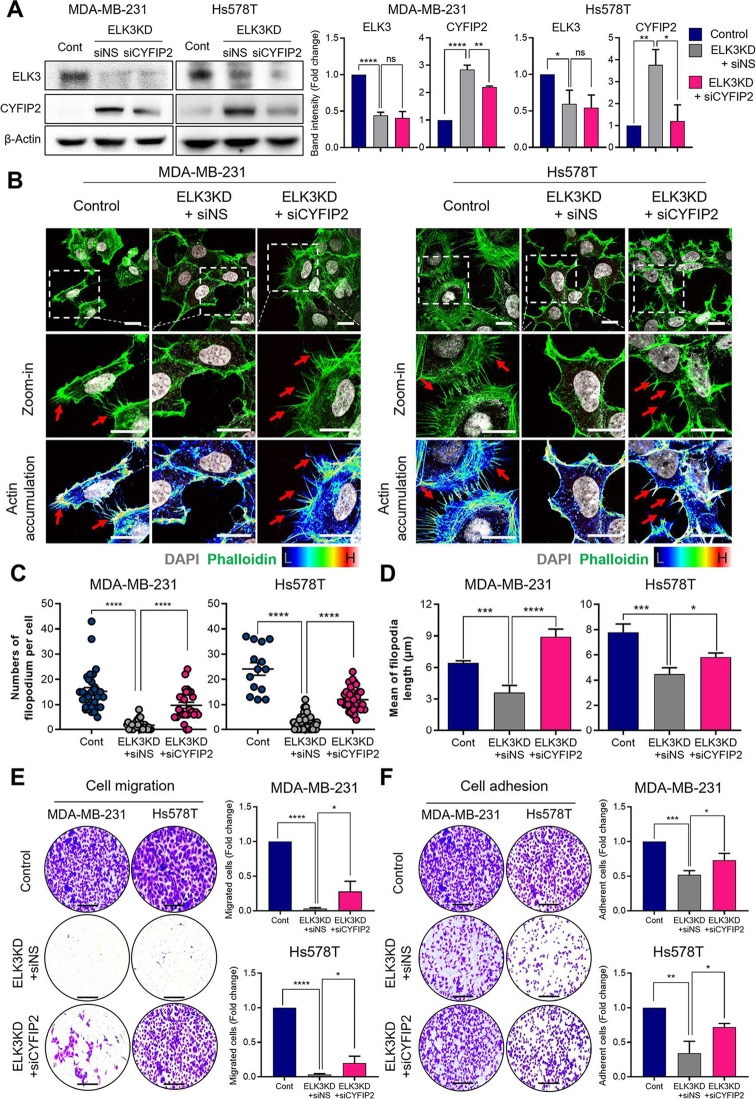
ELK3-CYFIP2 axis regulates metastatic nature of TNBCs by modulating flopodia protrusion. **A** Immunoblot analysis confrms the activity of siRNA targeting CYFIP2 (siCYFIP2) in ELK3KD MDA-MB-231 and Hs578T cells. ELK3KD TNBC cells transfected with a non-specifc siRNA (siNS) or siCYFIP2. Relative band intensity of ELK3 and CYFIP2. Data are presented as the mean ± SD. **B** Filopodia formation was observed after staining with DAPI and phalloidin. Actin accumulation of flopodia formation was visualized using fuorescence microscopy; representative protrusions are indicated by red arrows. Scale bar, 20 μm. **C** The number of flopodia per cell were quantifed, and is presented as individual dots. (MDA-MB-231 cells, *n* = 30, 30, and 26, respectively, Hs578T cells, *n* = 14, 30, and 30, respectively.) **D** The length of flopodia are presented in a graph. (MDA-MB-231 cells, *n* = 30, 30, and 26, respectively, Hs578T cells, *n* = 16, 30, and 30, respectively.) Data are presented as the SEM. **E–F** Representative images showing migration and adhesion of the indicated cells. Scale bar, 200 μm. All data were derived from at least three independent biological experiments. Data are presented as the mean ± SD. Control (Cont) = sh control of MDA-MB-231 or Hs578T cells; ELK3KD = ELK3KD of MDA-MB-231 or Hs578T cells. NS indicates no statistical signifcance. *P < 0.05; **P < 0.01; ***P < 0.001; ****P < 0.0001

## Correct Fig. 3

**Fig. 3:**
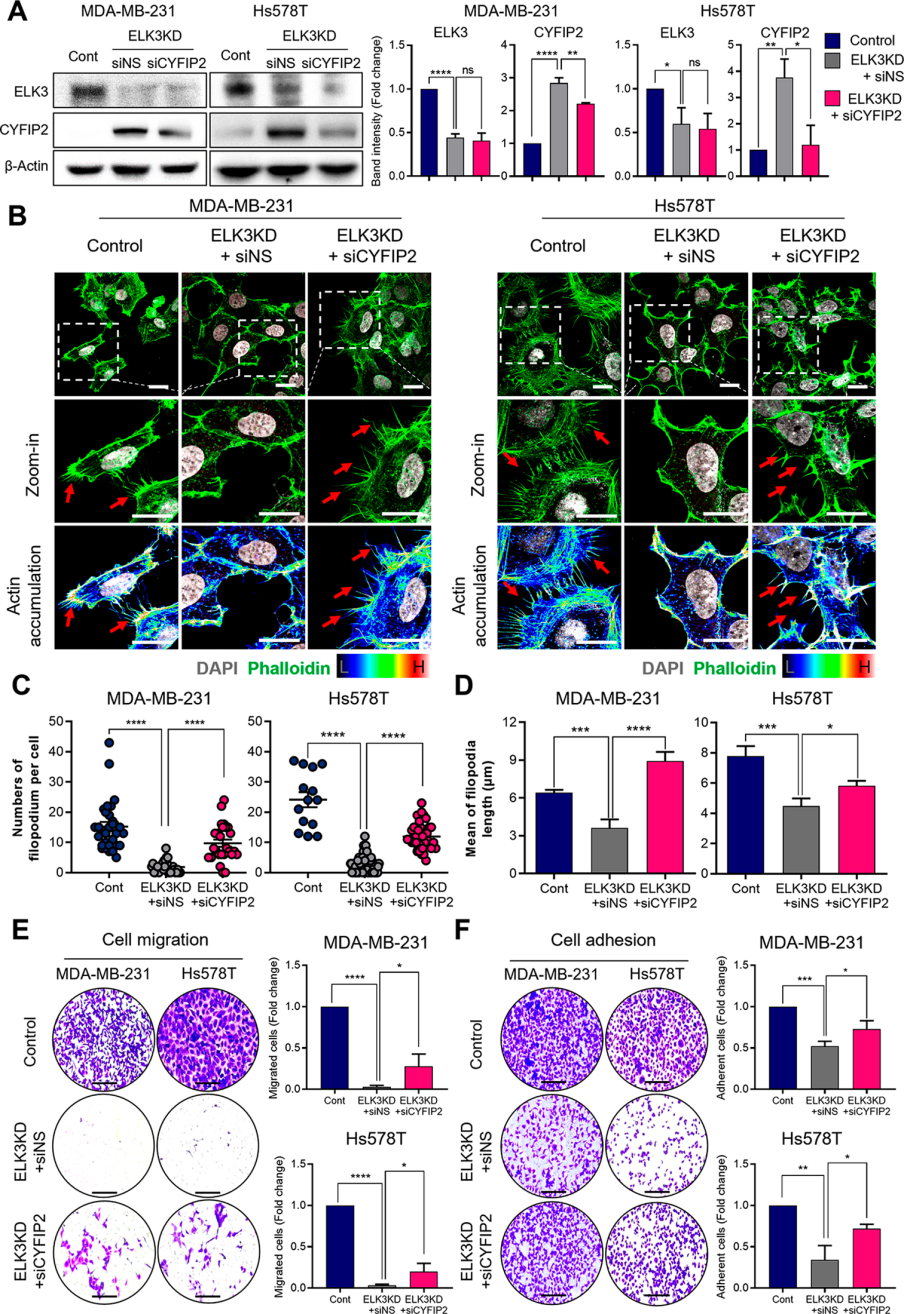
ELK3-CYFIP2 axis regulates metastatic nature of TNBCs by modulating flopodia protrusion. **A** Immunoblot analysis confrms the activity of siRNA targeting CYFIP2 (siCYFIP2) in ELK3KD MDA-MB-231 and Hs578T cells. ELK3KD TNBC cells transfected with a non-specifc siRNA (siNS) or siCYFIP2. Relative band intensity of ELK3 and CYFIP2. Data are presented as the mean ± SD. **B** Filopodia formation was observed after staining with DAPI and phalloidin. Actin accumulation of flopodia formation was visualized using fuorescence microscopy; representative protrusions are indicated by red arrows. Scale bar, 20 μm. **C** The number of flopodia per cell were quantifed, and is presented as individual dots. (MDA-MB-231 cells, *n* = 30, 30, and 26, respectively, Hs578T cells, *n* = 14, 30, and 30, respectively.) **D** The length of flopodia are presented in a graph. (MDA-MB-231 cells, *n* = 30, 30, and 26, respectively, Hs578T cells, *n* = 16, 30, and 30, respectively.) Data are presented as the SEM. **E–F** Representative images showing migration and adhesion of the indicated cells. Scale bar, 200 μm. All data were derived from at least three independent biological experiments. Data are presented as the mean ± SD. Control (Cont) = sh control of MDA-MB-231 or Hs578T cells; ELK3KD = ELK3KD of MDA-MB-231 or Hs578T cells. NS indicates no statistical signifcance. *P < 0.05; **P < 0.01; ***P < 0.001; ****P < 0.0001

The correction does not compromise the validity of the conclusions and the overall content of the article. The original article [1] has been updated.
