# Supplementary material for: ELK3-CYFIP2 axis-mediated actin remodeling modulates metastasis and natural killer cell responses in triple-negative breast cancer
Source: J Exp Clin Cancer Res. 2025 Feb 10;44:48. doi: 10.1186/s13046-025-03309-7 (PMC11808954; doi:10.1186/s13046-025-03309-7)
Supplement: Supplementary file 1 — Supplementary Material 1. [file 13046_2025_3309_MOESM1_ESM.docx]

**Supplemental information**

**Supplemental Figure**

**
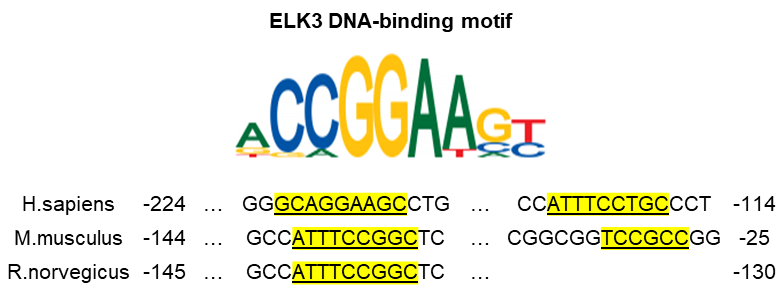
**

Supplemental Figure 1.

ELK3 binding motifs within the *CYFIP2* promoters of human, mouse, and rat. The ELK3 binding motifs are indicated in yellow.

**
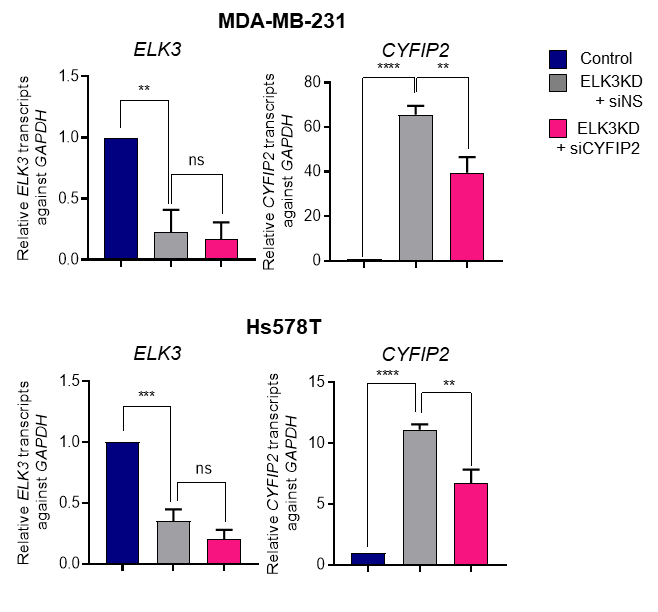
**

Supplemental Figure 2.

Effect of the ELK3-CYFIP2 axis on expression of ELK3 and CYFIP2 mRNA by control and ELK3KD TNBC cells. Quantitative RT-PCR confirms the activity of siRNA targeting CYFIP2 (siCYFIP2) in ELK3KD MDA-MB-231 and Hs578T cells. Control = sh control of MDA-MB-231 or Hs578T cells; ELK3KD = ELK3KD of MDA-MB-231 or Hs578T cells. Data represented as the mean ± SD. **P* < 0.05; ***P* < 0.01; ****P* < 0.001


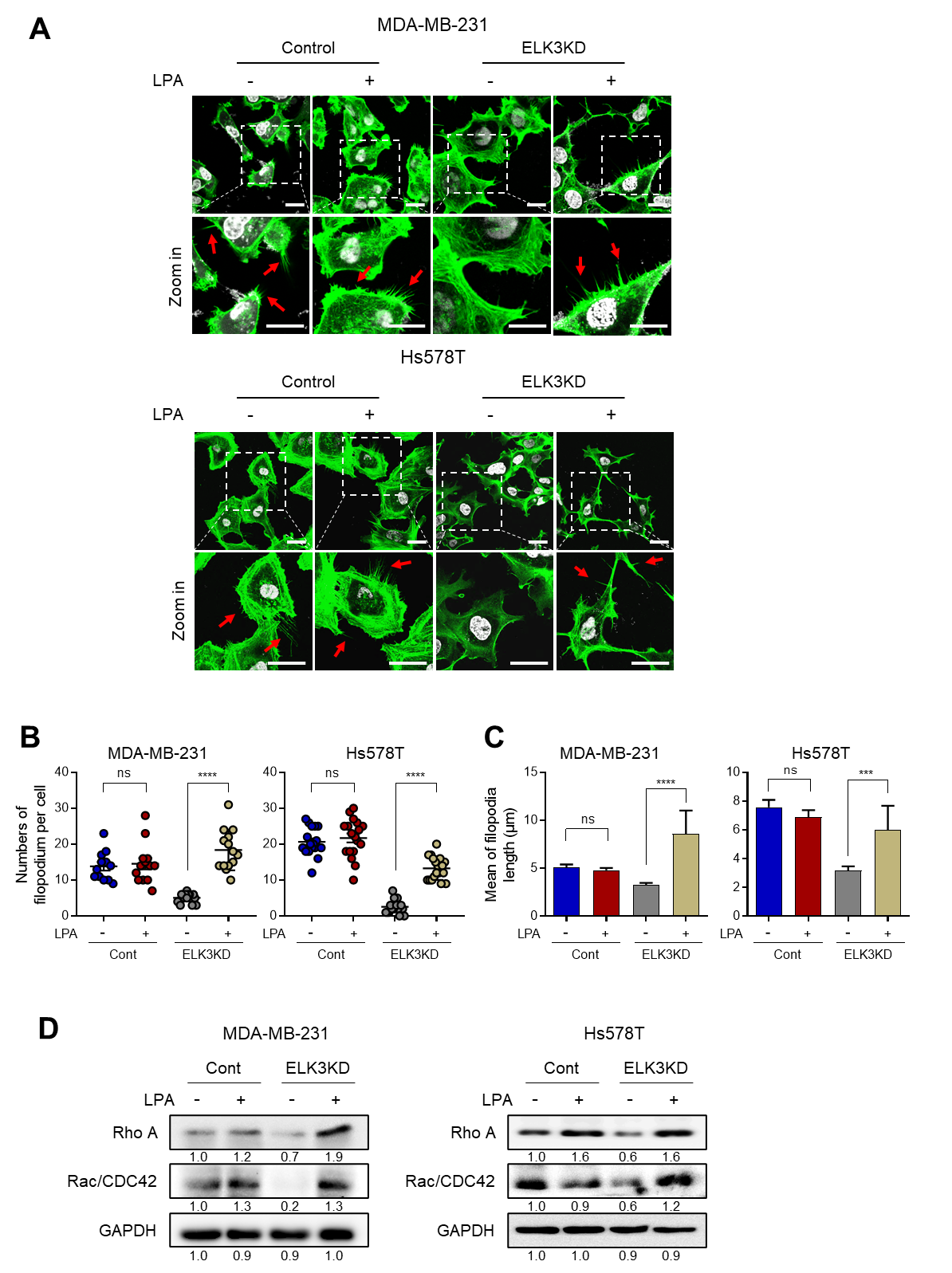


Supplemental Figure 3.

A Filopodia formation by control and ELK3KD MDA-MB-231 and Hs578T cells treated with LPA 10 µM for 24 h. Cells were stained with DAPI and phalloidin. Actin accumulation of filopodia formation was visualized using fluorescence microscopy. Scale bar, 20 µm. B The number of filopodia per cell were quantified and is presented as individual dots. (MDA-MB-231 cells, n=12, 13, 16 and 14 respectively, Hs578T cells, n=17, 18, 17 and 18 respectively) in the presence or absence of LPA. LPA was treated 10 µM for 24 h. C The length of filopodia are presented in a graph (MDA-MB-231 cells, n=12, 13, 16 and 14 respectively, Hs578T cells, n=15, 17, 15 and 18 respectively.) in the presence or absence of LPA. LPA was treated 10 µM for 24 h. D Immunoblot analysis confirms the downstream effects of Arp 2/3 signaling activation in control and ELK3KD (MDA-MB-231 and Hs578T) in the presence or absence of LPA. LPA was treated 10 µM for 24 h. Control (Cont) = sh control of MDA-MB-231 or Hs578T cells; ELK3KD = ELK3KD of MDA-MB-231 or cells. Data are presented as the SEM. **P* < 0.05; ***P* < 0.01; ****P* < 0.001


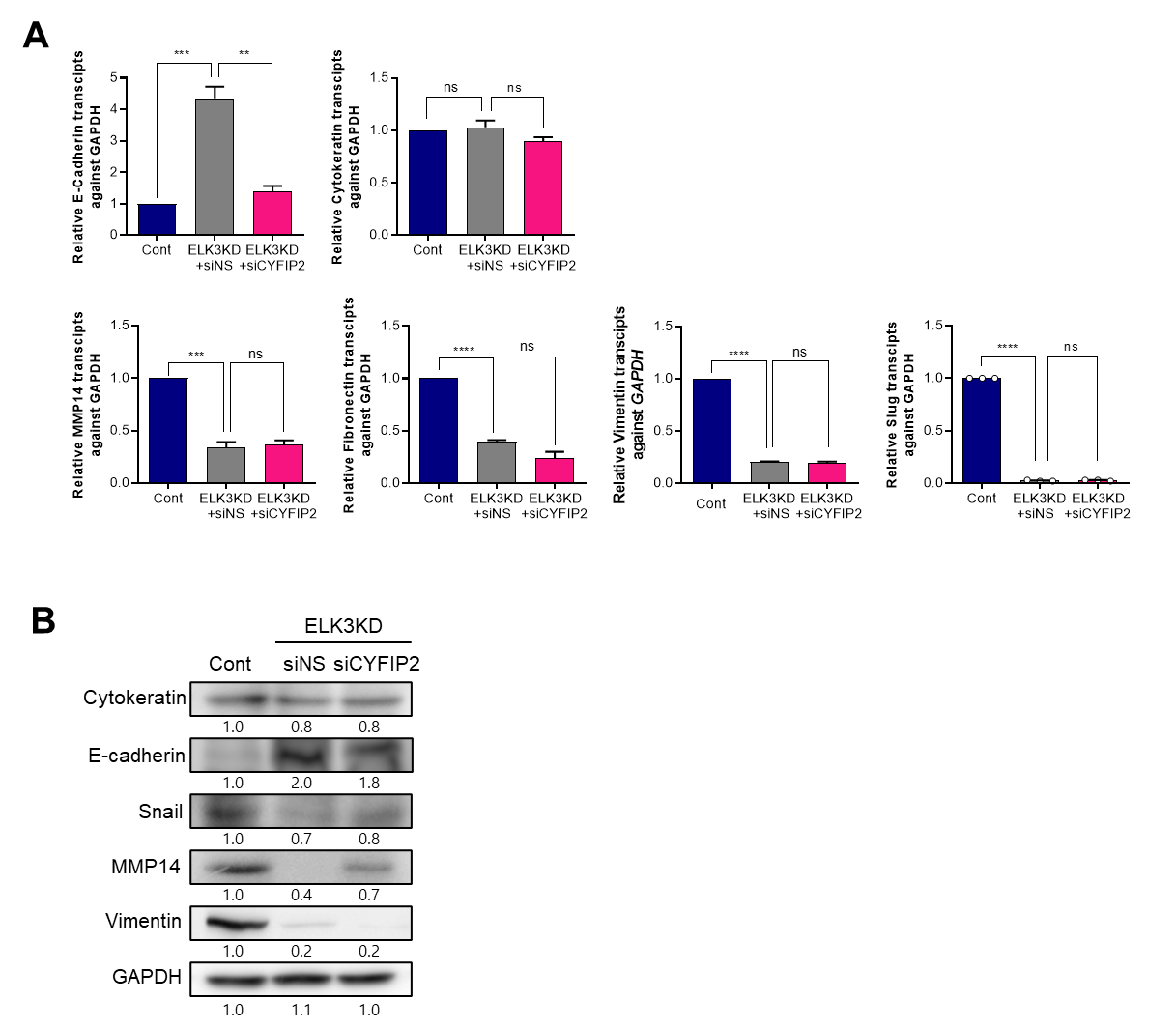


Supplemental Figure 4.

A Quantitative RT-PCR analysis showing relative mRNA expression levels of EMT markers. The expression levels of E-cadherin, Cytokeratin, MMP14, Fibronectin, Vimentin, and Snail were measured. Control (Cont) = sh control of MDA-MB- cells; ELK3KD = ELK3KD of MDA-MB-231 cells. Data represented as the mean ± SEM. ns = not significant; ***P* < 0.01; ****P* < 0.001; *****P* < 0.0001 B Western blot analysis of EMT-related proteins in control and ELK3KD cells treated with siNS or siCYFIP2. GAPDH was used as a loading control. The numbers below each band represent relative protein expression.

**
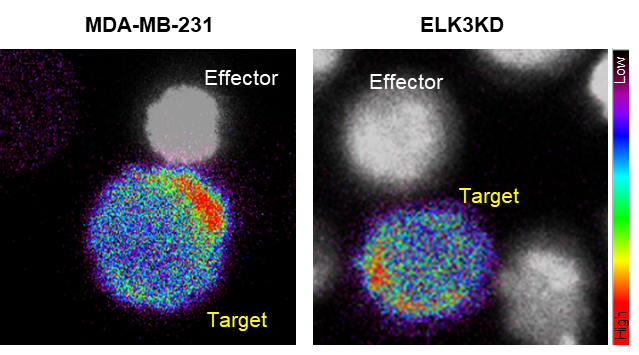
**

Supplemental Figure 5.

Movie showing responses of MDA-MB-231 or ELK3KD cells to NK-92MI cells (E:T ratio = 1:1). ELK3KD = ELK3KD of MDA-MB-231 cells.

**
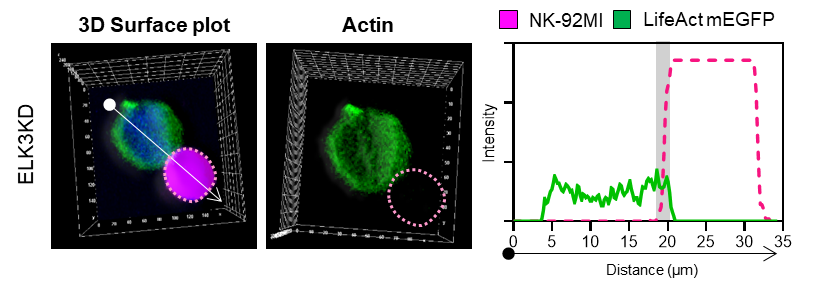
**

Supplemental Figure 6.

Actin responses were visualized to measure the intensity of actin accumulation at immunological synapses. ELK3KD = ELK3KD of MDA-MB-231 cells.


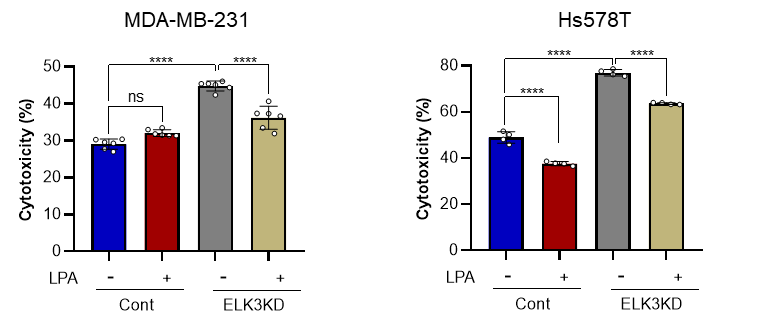


Supplemental Figure 7.

Immune response of control and ELK3KD MDA-MB-231 and Hs578T in the presence or absence of LPA. LPA was treated 10 µM for 24 h. The cytotoxic activity of NK-92MI against cancer cells was measured in a CFSE/7-AAD assay (E:T ratio = 10:1). All data were derived from at least three independent biological experiments. Data represented as the mean ± SD. Control (Cont) = sh control of MDA-MB-231 or Hs578T cells; ELK3KD = ELK3KD of MDA-MB-231 or Hs578T cells. Data represented as the mean ± SD. **P* < 0.05; ***P* < 0.01; ****P* < 0.001

**
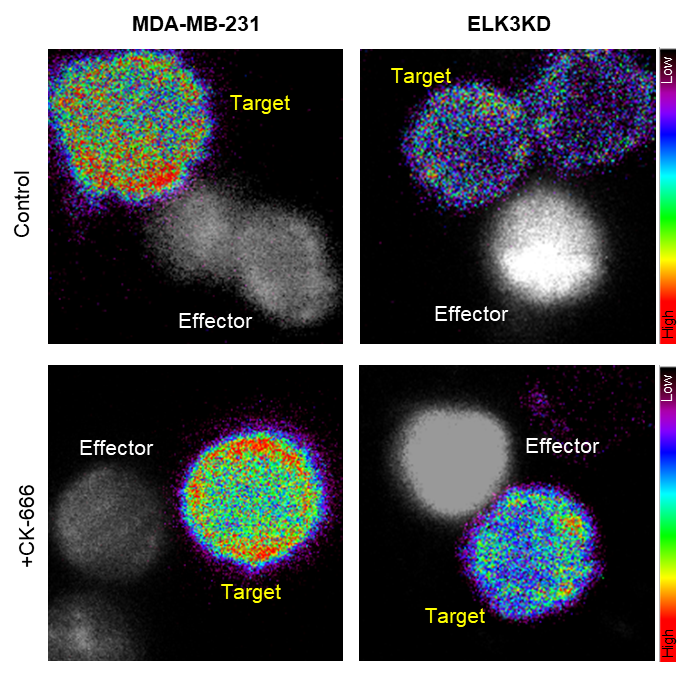
**

Supplemental Figure 8. Movie

Movie showing responses of MDA-MB-231 or ELK3KD cells to NK-92MI cells (E:T ratio = 1:1) in the presence of CK-666. Control = sh control of MDA-MB-231 cells; ELK3KD = ELK3KD of MDA-MB-231 cells.


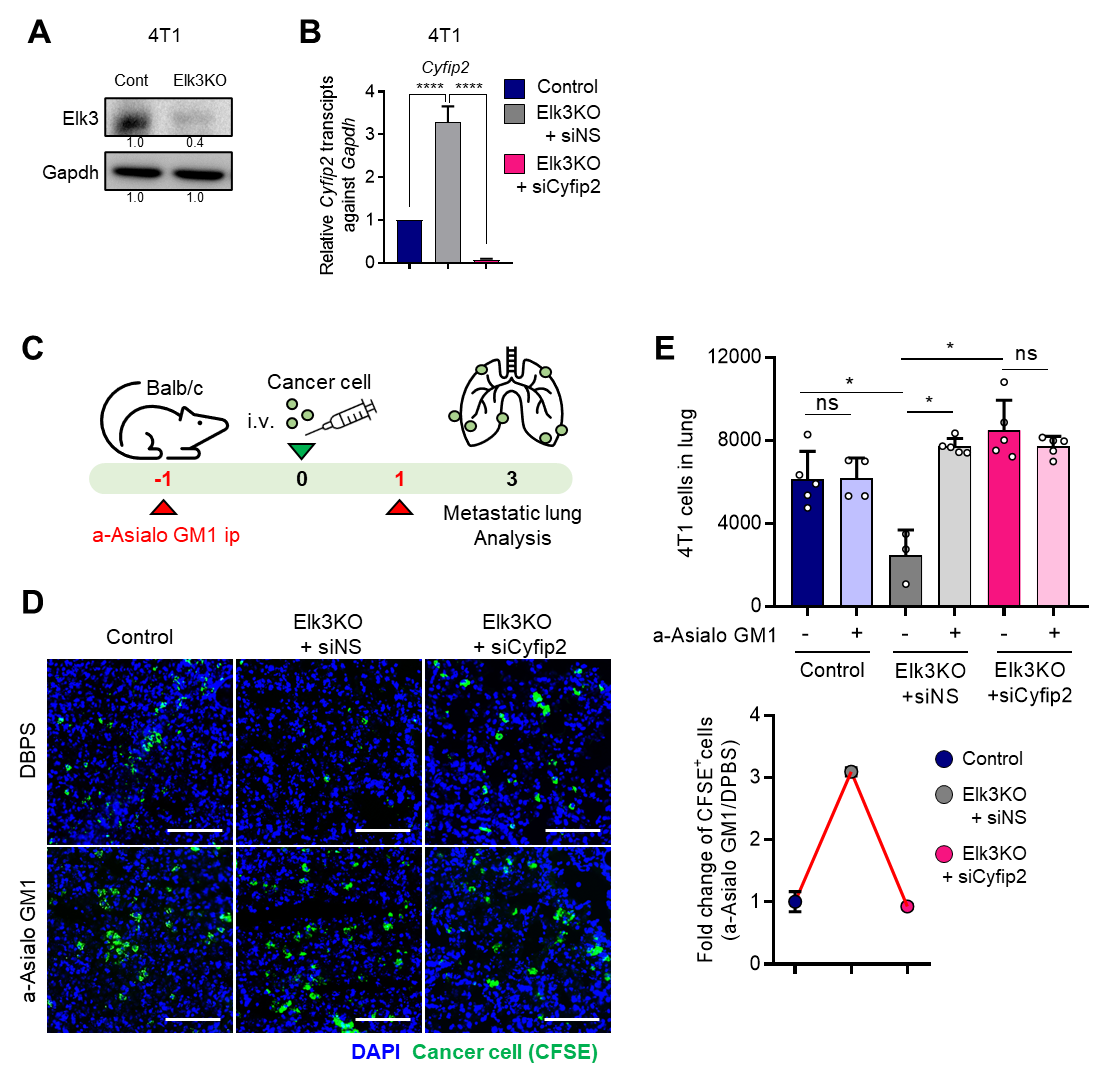


Supplemental Figure 9

ELK3-CYFIP2 axis-mediated regulation of metastasis and NK responses in a mouse model bearing 4T1 tumors. **A** Immunoblot analysis confirms the knockout of Elk3 in 4T1 cells. Control (Cont) = KO control of 4T1 cells; Elk3KO = Elk3 knockout of 4T1 cells. Elk3KO cells were treated with siNS or siCYFIP2. **B** Quantitative RT-PCR of confirms that Elk3 functions as a transcriptional repressor of Cyfip2 in mouse cell line and the activity of siRNA targeting Cyfip2 (siCyfip2) in 4T1 cells. **C** Schematic of the *in vivo* experiment: CFSE-labeled control, Elk3KO and Cyfip2-silenced Elk3KO 4T1 cells were injected intravenously into Balb/c mice. a-Asialo GM1 was injected intraperitoneally twice as indicated (each group n=3~5). **D** Fluorescence images of CFSE, indicating extravasated tumor cells in the lungs of mice at 3 days. Nuclei was stained with DAPI. Scale bar, 100 μm. **E** CFSE-positive cells in the lungs from each group of mice were quantified by flow cytometry. Data represented as the mean ± SD. **P* < 0.05; ***P* < 0.01; ****P* < 0.001

**Supplemental Table**

| **No** | **Name** | **Additional information** |
| --- | --- | --- |
| 1 | pLenti-cMyc-DDK | Origene, PS1000064 |
| 2 | pLenti-ELK3-cMyc-DDK | NM_005230.3 |
| 3 | pRL-TK | Promega, E2231 |
| 4 | pGL3-Basic | Promega, E1751 |
| 5 | pGL3-hCYFIP2 promoter (−1450 to 50 kb) | - |
| 6 | Nonspecific siRNA control | Bioneer, SN-1003 |
| 7 | hCYFIP2 siRNA | Bioneer, 26999-2 |
| 8 | mEGFP-Lifeact-7 | Addgene, 54610 |
| 9 | pCDH-EF1-MCS-T2A-Puro | Systembio, CD520A-1 |
| 10 | pCDH-EF1-MCS-mEGFP-Lifeact-7-T2A-Puro | - |

Supplemental Table 1. Plasmids and small interfering (si)RNAs used in the study

| **Genes** | **Forward Primer**  **(5' to 3')** | **Reverse Primer**  **(5' to 3')** | **Application** |
| --- | --- | --- | --- |
| *ELK3* | ACC CAA AGG CTT GGA AAT CT | TGT ATG CTG GAG AGC AGT GG | qRT-PCR |
| *CYFIP2* | CAA TCG CTA TGA AAC ACT GC | CCA GCG TCA TAT GCT TAC AG | qRT-PCR |
| *GAPDH* | GGG TGT GAA CCA TGA GAA | GTC TTC TGG GTG GCA GTG AT | qRT-PCR |
| *CYFIP2 Promoter (-264 to 95bp)* | GAG GCC GTG GAA GAA GCC T | GCG GCT GTC ACA CAA AGG G | ChIP-qPCR |

Abbreviations: ChIP, chromatin immunoprecipitation; qPCR, quantitative polymerase chain reaction.

**Supplemental Table 2. Primers used in the study**

| **Antibody** | **Manufacturer/supplier** | **Catalog No.** | **Application** |
| --- | --- | --- | --- |
| ELK3 | Novus Biologicals | NBP2-01264 | Immunoblot |
| CYFIP2 | Abcam | ab95969 | Immunoblot |
| β-Actin | Santa Cruz Biotechnology | sc-47778 | Immunoblot |
| Flag | MBL International Corporation | M185-3L | ChIP |

Abbreviations: ChIP, chromatin immunoprecipitation.

Supplemental Table 3. Antibodies used in the study
